# Design of new hole transport materials based on triphenylamine derivatives using different π-linkers for the application in perovskite solar cells. A theoretical study

**DOI:** 10.3389/fchem.2022.907556

**Published:** 2022-08-05

**Authors:** José David Quezada-Borja, Luz María Rodríguez-Valdez, Juan Pedro Palomares-Báez, Marco Antonio Chávez-Rojo, Linda-Lucila Landeros-Martinez, Mayra Cristina Martínez-Ceniceros, Gabriel Rojas-George, Isui Abril García-Montoya, Nora Aydeé Sánchez-Bojorge

**Affiliations:** ^1^ Facultad de Ciencias Químicas, Universidad Autónoma de Chihuahua, Circuito Universitario, Chihuahua, México; ^2^ CONACYT Research Fellow, Centro de Investigación en Materiales Avanzados (CIMAV), S.C., Miguel de Cervantes, Complejo Industrial Chihuahua, Chihuahua, México; ^3^ Departamento de Ciencias Químico-Biológicas, Instituto de Ciencias Biomédicas, Universidad Autónoma de Ciudad Juárez, Ciudad Juarez, Chihuahua, México

**Keywords:** triphenylamine (TPA), DFT-density functional theory, theoretical chemical reactivity, hole transport material (HTM), electronic properties

## Abstract

New organic molecules containing five different compounds, commonly called *p*-linkers, located between the triphenylamine units, were theoretically designed and analyzed in order to be proposed as new hole transport materials (HTMs) in perovskite solar cells, in total ten new molecules were analyzed. The electronic, optical and hole transport properties were determined, similarly, the relationship of these properties with their molecular structure was also investigated by Density Functional Theory (DFT) and Density Functional Tight Binding (DFTB) calculations. Eight of the ten analyzed compounds exhibited the main absorption band out of the visible region; therefore these compounds did not present an overlap with the absorption spectra of the typical methylammonium lead iodide (MAPI) hybrid-perovskite. The results showed that the Highest occupied molecular orbital (HOMO) levels of the compounds are higher than the perovskite HOMO level, and in some cases these are even higher than the Spiro-OMeTAD HOMO. The calculated electronic couplings and the reorganization energy values provided useful information in order to determine if the systems were hole or electron transport materials.

## Introduction

The depletion of fossil fuels reserves, growing energy demands, and the environmental crisis around the world have led the scientific community to search for new ways and technologies to use renewable energies sources efficiently. Due to the enormous amount of energy that the earth receives daily from the Sun, solar energy is considered the best choice among all renewable energy sources (Kim et al., 2020; Kumari et al., 2020; Pham et al., 2020; Sharmoukh et al., 2020). One of the most promising strategies to take advantage of solar energy is the conversion into electricity using photovoltaic devices (PV). Over the last few years, perovskite solar cells (PSCs), based on hybrid organic-inorganic halides, have achieved remarkable success as a result of their extraordinary optoelectronic properties such as their broad absorption region, direct band gap, high extinction coefficients, high charge carrier mobility, and long diffusion length (Mao et al., 2018; Mesquita et al., 2018; Ghaithan et al., 2020). PSCs offer low processing costs along with high energy conversion efficiency. These cells have the potential to replace silicon-based technology by requiring less material to obtain similar or larger power conversion efficiencies (PCE) (Zhou et al., 2018; Roy et al., 2020). Since the first report in 2009, the energy conversion efficiency of perovskite solar cells has increased from 3.81% to 25% in only a few years (Snaith, 2019; NREL, 2021). Hole transport materials play an important role in PSCs regenerating the oxidized state of perovskite and facilitating the hole transport (Pitchaiya et al., 2020; Lai et al., 2021). The importance of HTMs is not limited to charge transport, their properties stabilize the perovskite layer affecting the long-time performance of the cell (Zhang et al., 2018). Spiro-OMeTAD represents one of the most efficient HTMs to date and large efficiencies have been constantly reported using this compound (Li et al., 2021). However, the high costs of synthesis and its difficult purification have limited its commercialization (Sheibani et al., 2020). Therefore, the development of small, easy to prepare and low-cost production molecules has been an attractive target for many researchers (Gong et al., 2021). Resulting from their high hole mobility, thermal stability, and their non-crystalline or amorphous morphology, triphenylamine derivatives (TPAs) are HTMs that have been widely applied in organic electronic devices, such as OPVs, OLEDs, and OFETs (Rezaei and Mohajeri, 2021; Naeem et al., 2021). In the last few years, many research groups have developed different TPA-π-TPA HTMs by changing the π-linker due to the easy synthesis and the relatively simple structure of the compounds. The reported π-linkers include truxen (Wang et al., 2019), furan (Zhang et al., 2021a), triphenylamine (Kim et al., 2020), carbazole and fluorene (Leijtens et al., 2012), anthracene (Liu et al., 2017) pyrene (Gómez et al., 2021), silafluorene (Zhang and He, 2019), thiophene derivatives (Li et al., 2014; Zhou et al., 2020) to name a few. However, some of these materials show low power conversion efficiency, poor stability, or relatively high price. For these reasons, researchers have focused on finding new efficient and low-cost π-linkers for high-performance triphenylamine-based HTM. Pyrrole derivatives attract attention due to their low oxidation potential values (0.8 V) (Ansari, 2006), which are even lower than other heterocyclic compounds, such as thiophene which has values of 1.5 V (Camarada et al., 2011). This property and its wide use as hole transport material in organic light-emitting diodes (OLEDs) have favored the interest of expanding their application in perovskite solar cells. On the other hand, thiadiazoles have been used as polymers in PSCs, however, in the current work these molecules were used as π-linkers in the HTMs.

**FIGURE 1:**
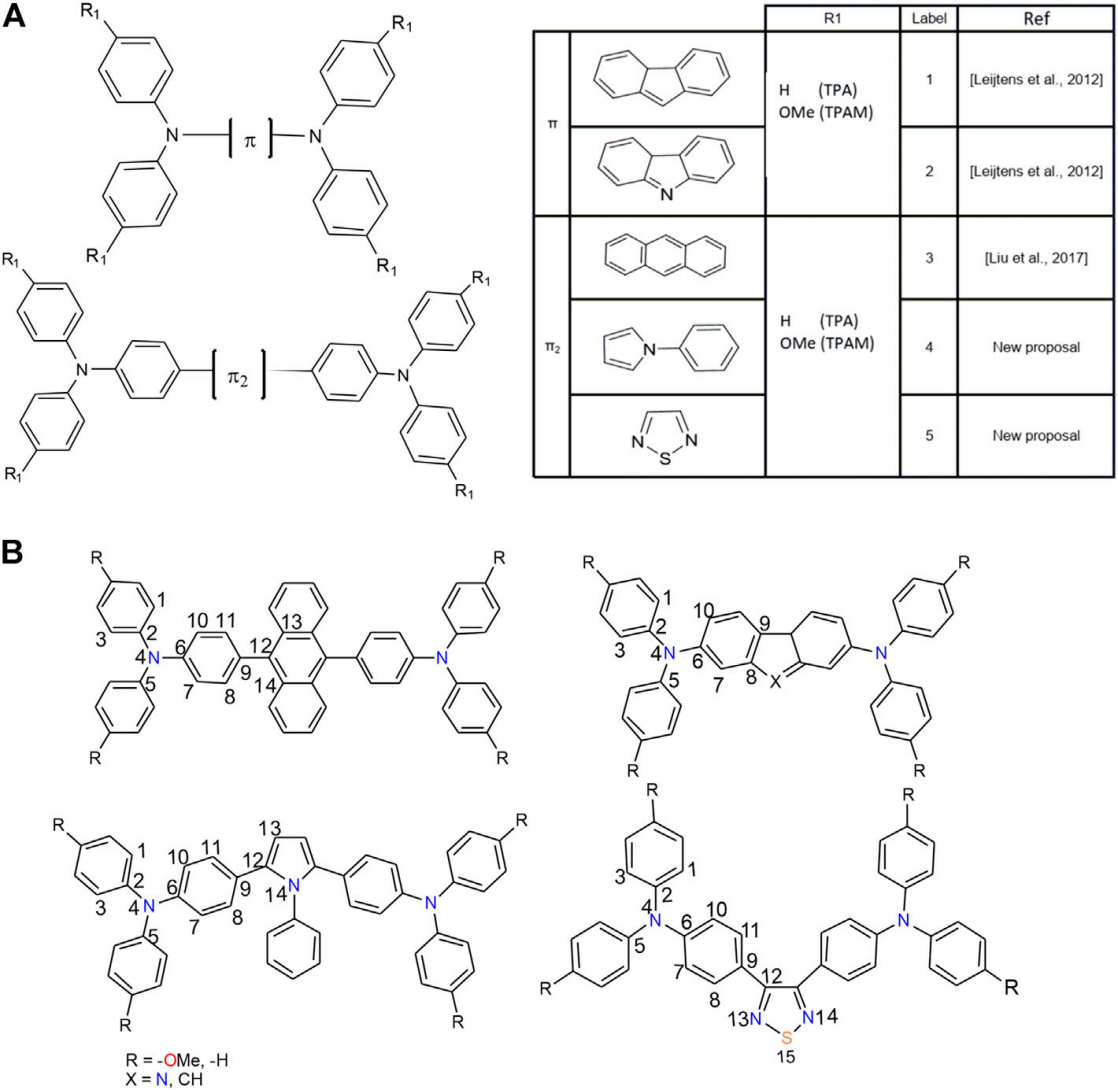
**(A)** Labeling scheme and general structure of the triphenylamine derivatives analyzed. **(B)** Numbering scheme for the atoms in the triphenylamine derivatives.

In the present study, new HTMs were proposed; these systems present a triarylamine-π-triarylamine architecture using pyrrole and thiadiazole as π-linkers (See Figure 1A). The electronic, optical and hole transport properties of these molecules were evaluated by Density Functional Theory (DFT) and Time Dependent-DFT. Also, the dimer of each molecule was constructed and these conformers were analyzed by Density Functional Tight Binding (DFTB) in order to obtain the intermolecular electronic coupling (transfer integral). The obtained results were compared with the reported properties of similar architectures based on fluorene, anthracene, and carbazole as π-linkers, as well as with the properties exhibited by Spiro-OMeTAD.

## Theoretical background and computational details

In the present work, the molecular geometries of the triphenylamine derivatives were optimized by the functional M06-2X (54% HF exchange) (Zhao and Truhlar, 2008) with the 6-311++G(d,p) basis set (Hariharan and Pople, 1973; Frisch et al., 1984). The functional M06-2X was selected because this functional has shown the best agreement with experimental results of infrared spectrum in previous work with carbazole derivatives (Zhang et al., 2016). All calculated geometries are in the global minimum of their potential energy surface; this was confirmed with frequency calculations.

The analyzed triphenylamine derivatives, were denoted as nTPA and nTPAM, where n corresponds to different π-linkers (1: Fluorene, 2: Carbazole, 3: Anthracene, 4: Pyrrole and 5: Thiadiazole), TPA refers to triphenylamine units and M represents the presence of the methoxy groups in the structure (see Figure 1A).

In order to obtain the best accuracy with the experimental maximum absorption wavelength results, the already synthesized: Fluorene-TPA (1TPA), carbazole-TPA (2TPA), and anthracene-TPA (3TPAM), were analyzed using TD-DFT (Bartolotti, 1982; Runge and Gross, 1984). Several functionals: B3LYP (London, 1937), M06-L (Zhao and Truhlar, 2006), M06 (Zhao and Truhlar, 2008), M06-2X (Zhao and Truhlar, 2008) and PBE (Adamo and Barone, 1999), wB97XD (Chai and Head-Gordon, 2008), CAM-B3LYP (Yanai et al., 2004) in combination with 6-311++G(d,p) basis set, were applied for the analysis. All the calculations were carried out using chlorobenzene as solvent, with the solvation model based on density method (SMD) (Marenich et al., 2009). The TD-M06/6-311++G(d,p) was selected as the best methodology for the determination of absorption properties.

The chemical reactivity parameters, such as ionization potential (IP), electron affinity (EA), and chemical hardness (*η*) were calculated using DFT M06/6-311++G(d,p) The definitions of these chemical reactivity parameters can be written as:

EA=E(N)−E(N+1) (1)

IP=E(N−1)−E(N) (2)

$$\eta =\frac{IP-EA}{2}$$ (3)

Where E(N) , E(N+1) and E(N−1) are the energy of the molecules with N, N+1 and N-1 electrons. The electronic properties like HOMO (Highest occupied molecular orbital) and LUMO (lowest unoccupied molecular orbital) energy, as well as the spatial distribution of the molecular orbitals related to the principal electronic transitions, were calculated with M06/6-311++G(d,p). The functional M06 was selected because some studies showed that functionals with small Hartree-Fock exchange are more appropriate for electronic calculations. (Dev et al., 2012; Delgado-Montiel et al., 2016; Rostami et al., 2018). All the calculations were made considering the approximation of isolated molecules. See Scheme 1 in supplementary material.

**FIGURE 2:**
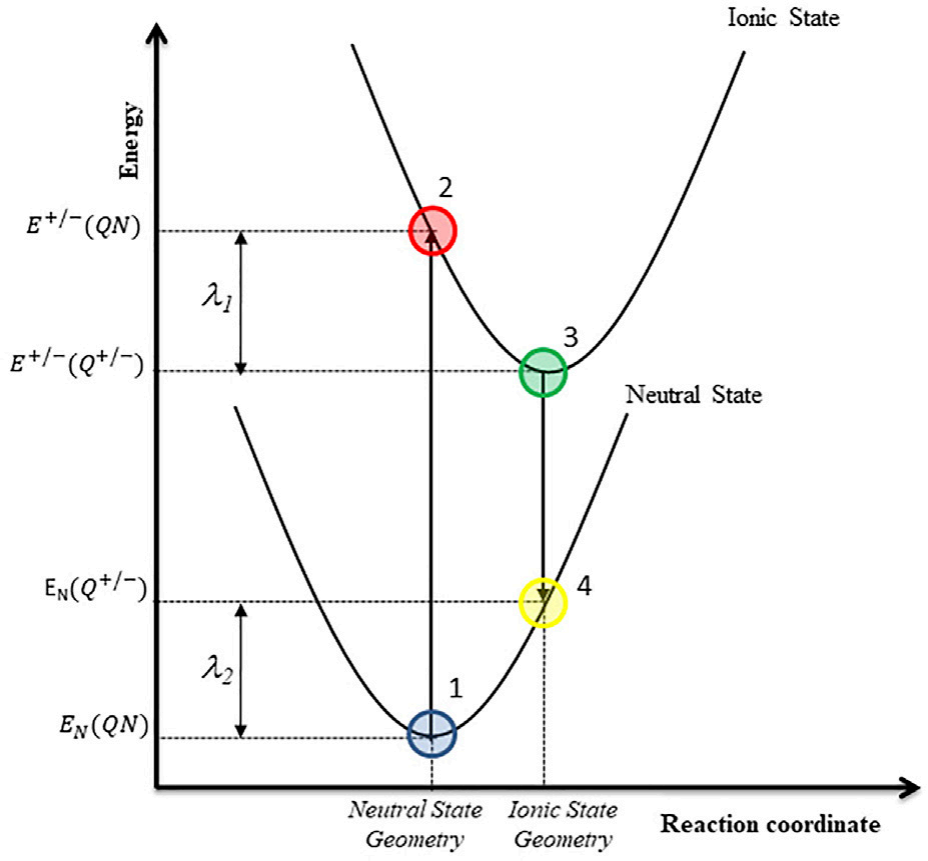
Four-point model used for the reorganization energy calculations.

In all the analyzed compounds, the internal reorganization energy, for holes and electrons, was calculated with M06/6-31G(d) level of theory. The charge transport in each compound was calculated based on Marcus’ theory. This theory is based on the assumption that charges are located in a single molecule and charge transfer reactions take place through a jump mechanism between sites (Marcus, 1993). According to this, the carrier transport rate between a pair of molecules at a fixed temperature is expressed as:

$$K_{et}=\frac{4\pi^{2}}{h}\frac{t^{2}}{\sqrt{4\pi \lambda k_{B}T}}exp\left(-\frac{\lambda }{4k_{B}T}\right)$$ (4)

where *T* is the absolute temperature, *t* is the charge transfer integral, *k*_*B*_ and *h* are the Boltzmann and Planck constant respectively, and *λ* is the reorganization energy. The latter is the sum of the energies associated to the rearrangements of the molecular geometry when: i) the neutral state is ionized vertically to the cationic/anionic state and ii) the stable cationic/anionic state returns to the neutral state. Nelsen et al. (1987) have provided a convenient way to calculate the reorganization energy using a four-point model, generally called the adiabatic potential energy surface method (Nelsen et al., 1987; Touhami et al., 2015), which is illustrated in Figure 2.

The reorganization energy for the hole (λ^**+**^) and electron (λ^**-**^) transfer was calculated with the Eqs 5–7.

$$\lambda_{T}^{+/-}=\lambda_{1}^{+/-}+\lambda_{2}^{+/-}$$ (5)

$$\lambda_{1}^{+/-}=\;E^{+}\left(Q_{N}\right)-\;E^{+}\left(Q^{+}\right)$$ (6)

$$\lambda_{2}^{+/-}=\;E_{N}\left(Q^{+}\right)-\;E_{N}\left(Q_{N}\right)$$ (7)

Where $\lambda_{1}^{+/-}$ is calculated with the difference between $E^{+/-}\left(Q_{N}\right)$ and $E^{+/-}\left(Q^{+/-}\right)$ . The first is the cationic E^+^(Q_N_) and anionic E^−^(Q_N_) energy after a vertical ionization of a neutral molecule. While $E^{+/-}\left(Q^{+/-}\right)$ symbolizes the total energy of the optimized cationic structure $E^{+}\left(Q^{+}\right)$ and optimized anionic structures $E^{-}\left(Q^{-}\right)$ after a geometric relaxation. Similarly, $\;\lambda_{2}^{+/-}$ is calculated with the difference between $E_{N}\left(Q^{+/-}\right)$ and $E_{N}\left(Q_{N}\right)$ . Where $E_{N}\left(Q^{+/-}\right)$ is the total energy after a vertical neutralization of a charged molecule, and $E_{N}\left(Q_{N}\right)$ symbolizes the energy of neutral optimized geometry. All above-mentioned calculations were performed by the Gaussian 09 program in gas phase (Gaussian 09, 2010).

The intermolecular electronic coupling (transfer integral) is another key parameter to calculate the charge transfer rate. The transfer integrals were calculated using the *dimer projection method* (Valeev et al., 2006; Baumeier et al., 2010) as implemented in the CATNIP software (Brown, 2022). In this method, the electronic coupling between donor and acceptor (molecules A and B) is given by,

$$J_{AB}^{eff}=t_{AB}=\frac{J_{AB}-\frac{1}{2}\left(e_{A}-e_{B}\right)S_{AB}}{1-S_{AB}^{2}}$$ (8)

where $J_{AB}$ , $S_{AB}$ and $e_{A\left(B\right)}$ (charge transfer integrals, spatial overlap and the site energies in the non-orthogonal frontier orbital basis) were calculated using the following equations:

$$\begin{array}{l} e_{A}=\;\langle \phi^{A}|H|\phi^{A}\rangle ,\\ e_{B}=\;\langle \phi^{B}|H|\phi^{B}\rangle ,\\ J_{AB}=\;\langle \phi^{A}|H|\phi^{B}\rangle ,\\ S_{AB}=\;\langle \phi^{A}|\phi^{B}\rangle . \end{array}$$ (9)

Here, $\phi^{A\left(B\right)}$ are the frontier orbitals of the molecules A and B, and H , is the Kohn-Sham Hamiltonian of the dimer system. The electronic coupling calculations were carried out at the B3LYP/6-311+G(d) level of theory. In recent studies it was reported that this methodology showed a good correlation with data reference (Ziogos et al., 2021).

The dimers of every molecule were built using a Montecarlo optimization algorithm at zero temperature as described below. First, the coordinates of the original (optimized) monomers were duplicated to produce a second monomer at a distance equal to twice the length of the molecule. Second, the molecule was rotated randomly in order to generate the starting configuration. A series of translations were then performed on the second monomer coordinates keeping both molecules as rigid bodies. After every translation, the total intermolecular (atom-atom) Lennard-Jones potential energy was calculated in order to accept only those displacements that minimized the energy. Once stabilized, the second molecule was allowed to perform rotations looking for the minimum energy configuration. This procedure was repeated hundreds of times, starting from different initial positions and configurations. The structure obtained by this method was further optimized using the DFTB (Density Functional based Tight Binding) method, at the DFTB-3DBJ level, as implemented in the DFTB + software (Hourahine et al., 2020).

## Results and discussion

### Geometrical structure

**FIGURE 3:**
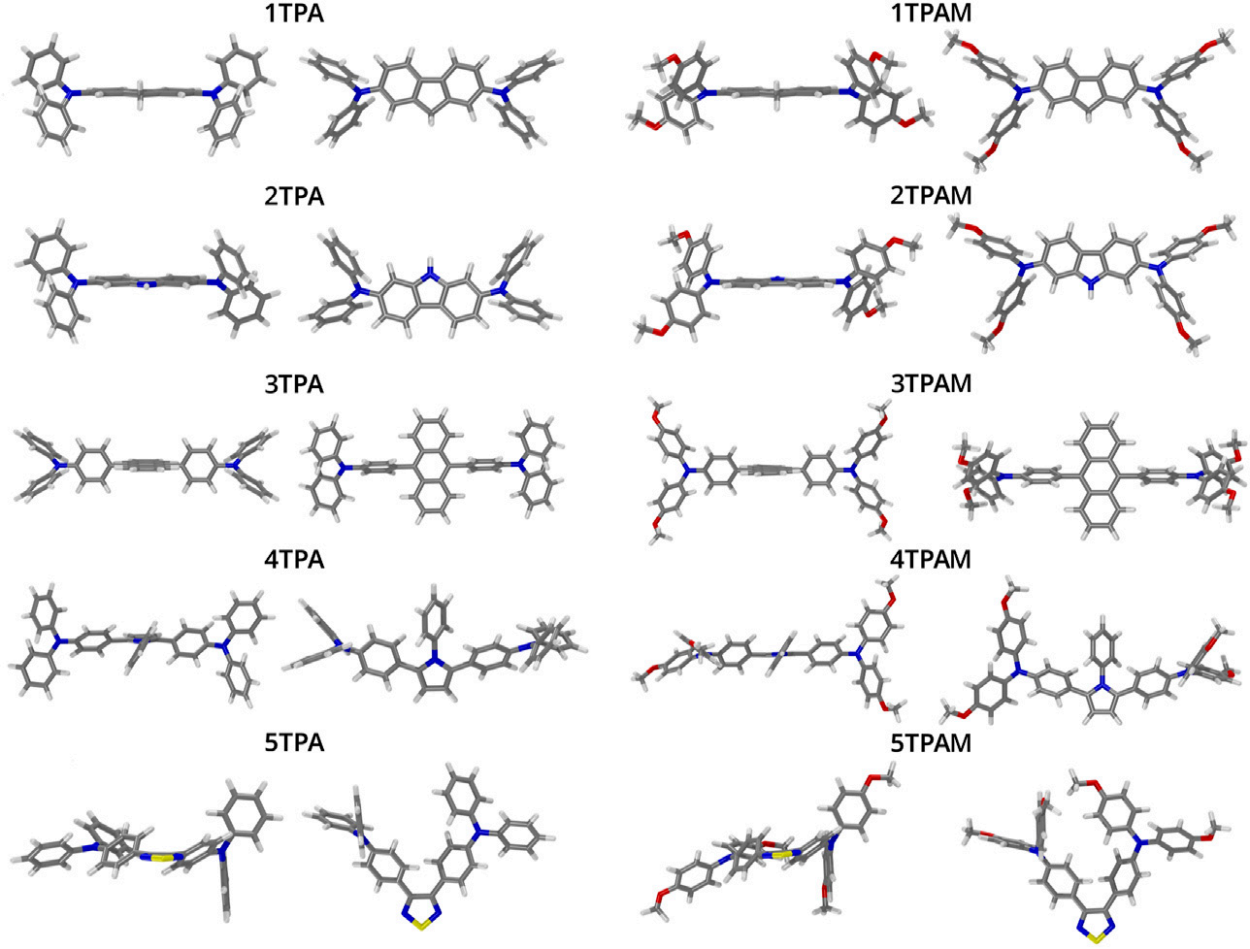
Optimized geometrical structures of the triphenylamine derivatives obtained with M06-2X/6-311++G(d,p).

The optimized structures of triphenylamine derivatives at the M06-2X/6-311++G(d,p) level of theory are shown in Figure 3. The vibration frequencies were calculated at the same level of theory to verify that the stationary points correspond to the global minimum of the potential energy hypersurface. The compound 4TPA exhibited an almost similar structure to those compounds that incorporate carbazole and fluorene as π-linker, therefore, it can be expected that its properties were similar to the carbazol and fluorene derivatives. 4TPA presented a nearly planar structure, mainly in the region of the π-linker, this planarity gives more uniformity to the electronic density, for this reason it is expected the charge transport to be promoted.

On the other hand, some derivatives showed torsion angles between π-linker and triphenylamine units, which could affect the charge transport process. Table 1 lists the geometrical parameters for all the systems, such as angles and torsion angles related to π-linker, and Figure 1B shows the used labels.

**TABLE 1:** Geometrical parameters for all triphenylamine derivatives calculated with M06-2X/6-311++G(d,p).

Atom/Compound Angle (°)	TPA					TPAM				
	1	2	3	4	5	1	2	3	4	5
**C1-C2-N4**	120.2	120.3	120.3	120.3	120.5	120.5	120.7	121.0	121.1	121.4
**C3-C2-N4**	120.5	120.4	120.5	120.3	120.1	120.8	120.7	120.3	120.2	119.8
**C2-N4-C6**	119.9	119.5	119.9	120.0	120.9	120.3	120.5	120.6	120.5	119.0
**C5-N4-C6**	119.8	120.0	119.9	120.3	119.8	120.4	119.9	120.6	120.4	119.8
**N4-C6-C10**	120.2	119.3	120.5	120.7	121.0	120.3	119.4	120.7	120.6	119.4
**N4-C6-C7**	120.0	120.0	120.5	120.5	120.1	120.1	120.1	120.7	120.8	121.6
**C11-C9-C12**	—	—	120.9	119.1	120.0	—	—	121.0	119.4	120.4
**C9-C12-C13**	—	—	120.0	127.9	120.1	—	—	120.0	128.1	121.9
**C8-C9-C12**	—	—	120.8	122.9	121.3	—	—	121.0	122.8	120.6
**C9-C12-C14**	—	—	120.0	124.6		—	—	120.0	124.3	—
Dihedral Angle **(°)**										
**C2-N4-C6-C10**	−39.1	−45.3	40.0	35.6	33.2	32.6	−43.0	32.4	34.9	43.7
**C2-N4-C6-C7**	141.3	134.8	140.0	−144.6	−147.1	−147.6	137.4	−147.5	−145.2	−134.7
**C5-N4-C6-C10**	136.5	138.8	140.0	−141.4	−144.5	−144.0	143.8	−147.5	−148.2	−152.9
**C5-N4-C6-C7**	−43.0	−40.9	40.0	38.3	35.0	35.8	−35.7	32.4	31.4	28.5
**C11-C9-C12-C13**	—	—	−74.0	42.0	136.5	—	—	−106.6	44.1	131.4
**C11-C9-C12-C14**	—	—	106.0	−141.6	—	—	—	73.3	−139.3	—
**C8-C9-C12-C14**	—	—	−73.9	41.4	—	—	—	−106.6	43.3	—
**C8-C9-C12-C13**	—	—	106.0	−134.8	−42.5	—	—	73.4	-133.1	-53.4

The torsion angles analysis was carried out through the comparison of the same torsion angle present in different derivatives. The 3, 4, 5TPA and 3, 4, 5TPAM derivatives showed a torsion in C11-C9-C12-C13 atoms due to the bond type C—C between the triphenylamine unit and π-linker. As can be observed, 5TPA and 5TPAM exhibited the greatest values of torsion angles. This behavior is due to the inclusion of a thiadiazole heterocycle in the structure. Further, a difference was observed in the torsion angles of 3TPA and 3TPAM molecules; this difference could be attributed to the effect of the methoxy group in the triphenylamine unit. It was observed that the addition of methoxy groups in 3TPA and 3TPAM breaks the planarity of the molecules. Nevertheless, 4TPA and 4TPAM derivatives did not show a significant difference in the torsion angle values, which denote that the molecules with a benzopyrrole unit are not affected when methoxy groups are added. This similar behavior is observed in 1TPA, 1TPAM, 2TPA, and 2TPAM.

A significant difference was observed in the torsion angle C2-N4-C6-C1O only between 5TPA and 5TPAM, which registered a difference of 10.5°, however, in the other compounds, this torsion angle did not show significant changes because it is part of the triphenylamine rings. This difference in the torsion angle (5TPA and 5TPAM) could also be ascribed to inclusion of methoxy groups in derivatives with thiadiazole as π-linker, which could affect the planarity of the molecule.

### Electronic properties

One of the most significant characteristics of HTMs is to be transparent to visible light when an inverse cell is used, to be precise, these must not absorb energy in the visible region of the electromagnetic spectrum (400–700 nm). Therefore, several functionals were probed to determine the electronic properties of triphenylamine derivatives. The comparison between calculated maximum absorption wavelengths and the reported experimental values was used as a criterion to select the employed methodology.

**FIGURE 4:**
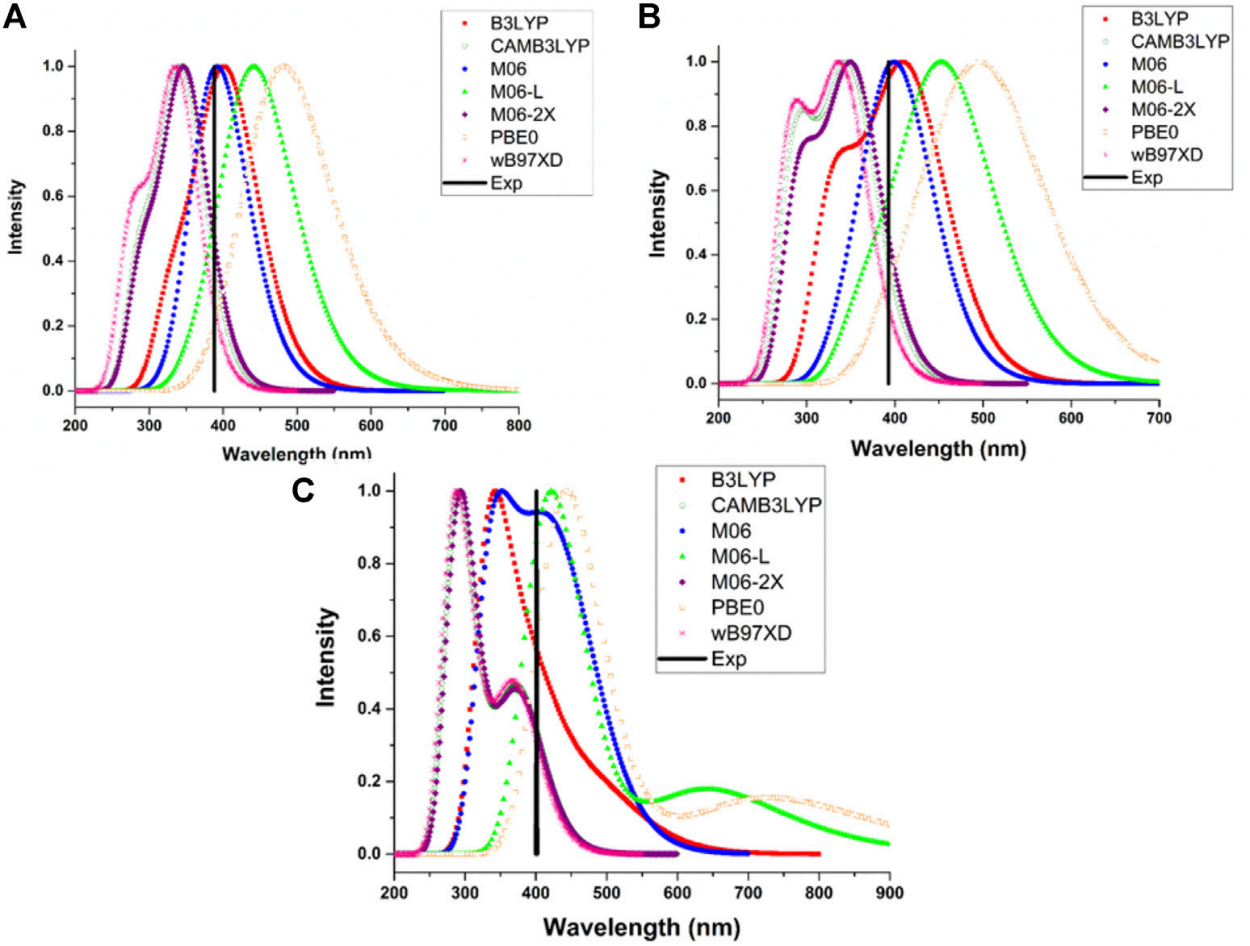
Comparison of the experimental maximum absorption wavelength with the calculated absorption spectra for the **(A)** 1TPAM, **(B)** 2TPAM and **(C)** 3TPAM, obtained with several functionals.

The theoretical maximum absorption wavelengths for 1, 2, 3TPAM calculated with several functionals and their corresponding experimental values are shown in Table 2 and Figure 4. The results obtained with M06 functional agree with experimental absorption peaks, where a slight difference was observed (less than 18 nm) between the calculated and reported values for carbazole (AS37) and fluorene (AS44) derivatives (Leijtens et al., 2012). Nevertheless, only for the anthracene derivative, the obtained value with M06 functional showed a difference of 62 nm when compared to the reported value (Liu et al., 2017). On the other hand, some studies have reported that the M06 functional describes more precisely the absorption spectra for compounds with a high degree of delocalization (Soto-Rojo et al., 2015). For this reason, the M06 functional in combination with 6-311++G(d,p) basis set, was selected to calculate the absorption properties of the new compounds (pyrrole and thiadiazole derivatives).

**TABLE 2:** Maximum absorption wavelength for 1, 2, and 3TPAM calculated with different funcionals and 6-311++G(d,p) basis set; and the comparison with the experimental value.

Compound/Methodology	*λ (nm)*							*λ*_exp_ (*nm*)
	B3LYP	CAMB3LYP	M06	M06-L	M06-2X	PBE	wB97XD	
1TPAM	416.3	351.0	406	435	354	477	342	388 Leijtens et al. (2012)
2TPAM	418	341	406	464	355	514	343	393 Leijtens et al. (2012)
3TPAM	338	296	339	424	300	445	301	401 Leijtens et al. (2012)

**FIGURE 5:**
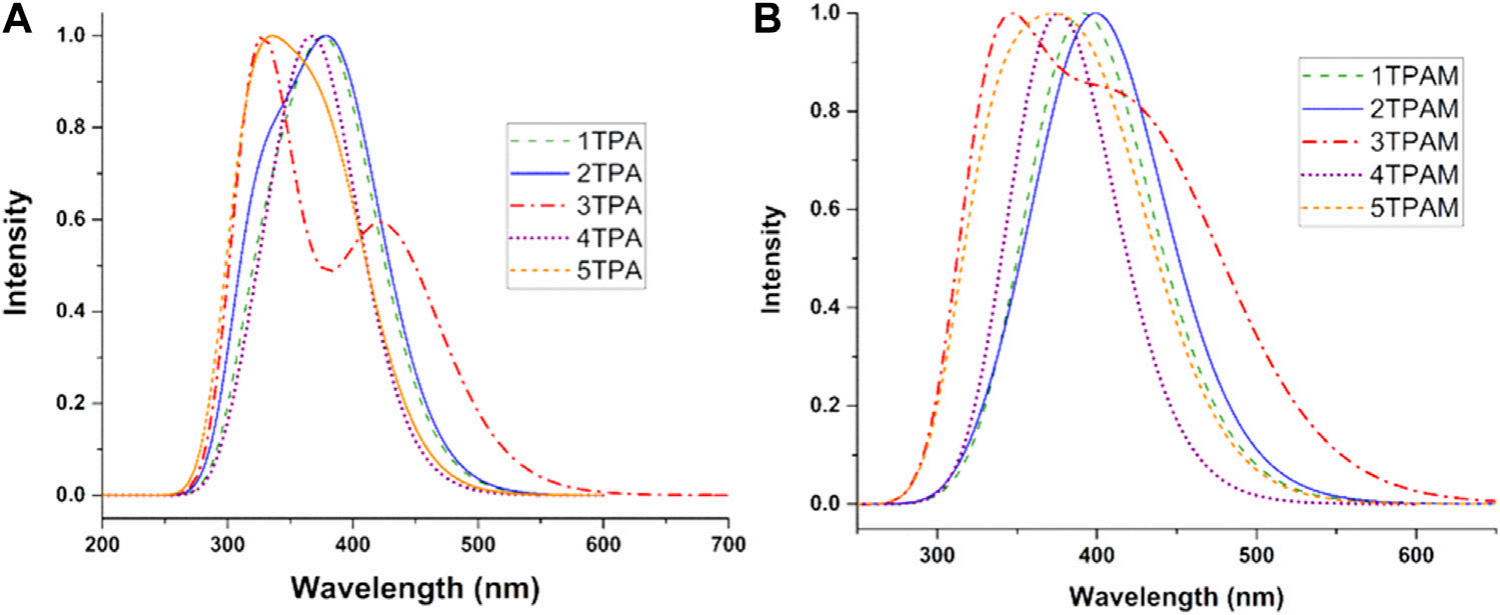
Absorption spectra of the ten analyzed triphenylamine derivatives calculated with M06/6-311++G(d,p), using chlorobenzene as solvent and employing SMD solvation model. **(A)** R = H and **(B)** R = OMe.

TD-DFT calculations were performed at the M06/6-311++G(d,p) level of theory using chlorobenzene as a solvent and employing SMD solvation model (Marenich et al., 2009) to simulate the absorption properties of the triphenylamine derivatives. The absorption spectra of all the analyzed compounds are shown in Figure 5. Table 3 shows the theoretical results for electronic transitions of the triphenylamine derivatives: maximum absorption wavelengths (λ_max_), vertical absorption energy (Ω_A_), oscillator strength (*f*), and the electronic transitions corresponding to each chemical system. As can be observed in Figure 5 all the compounds presented a minimal or null absorption in the visible region, except the anthracene derivative (3TPA and 3TPAM). Because of this, almost all the compounds could be used in a PSC, since the absorption spectra of the derivatives and the MAPI perovskite are not overlapping, which avoids a loss of efficiency due to the charge recombination (Gheno et al., 2016; Ortiz, 2019). Additionally, the absorption spectrum of pyrrole derivatives showed similar behavior to carbazole and fluorine derivatives, mainly in the TPA derivatives.

**TABLE 3:** Maximum absorption wavelengths (*λ*), vertical absorption energy (Ω_A_), oscillator strength (*f*) and the main electronic transitions of triphenylamine derivatives calculated with M06/6-311++G(d,p).

Compounds	*λ (nm)*	$\Omega_{A}\left(eV\right)$	Oscillator strenght (f)	Electronic transitions
1TPA/1TPAM	387.6/406.8	3.20/3.05	0.9384/0.5856	H-0→L+0(+90%)/H-0→L+0(+52%)
	362.3/388.1	3.42/3.20	0.1658/0.4646	H-0→L+1(+58%)/H-0→L+2(+41%)
	329.0/372.4	3.77/3.33	0.4177/0.2173	H-0→L+9(+55%)/H-0→L+6(+64%)
2TPA/2TPAM	388.2/406.1	3.19/3.05	0.8796/0.8399	H-0→L+0(+94%)/H-0→L+1(+82%)
	328.3/372.6	3.78/3.33	0.1963/0.1268	H-0→L+7(+60%)/H-0→L+2(+59%)
	328.8/355.1	3.77/3.49	0.1326/0.0589	H-0→L+3(+21%)/H-0→L+9(+39%)
3TPA/3TPAM	427.2/450.5	2.90/2.75	0.3844/0.2398	H-0→L+0(+87%)/H-0→L+0(+93%)
	327.6/339.2	3.78/3.66	0.5874/0.3280	H-0→L+3(+26%)/H-0→L+4(+37%)
	322.0/399.6	3.85/3.10	0.0748/0.2097	H-1→L+1(+24%)/H-2→L+0(+92%)
4TPA/4TPAM	373.3/380.0	3.32/3.26	1.3138/0.6177	H-0→L+1(+63%)/H-0→L+2(+25%)
	327.9/376.4	3.78/3.29	0.1962/0.5861	H-0→L+4(+39%)/H-0→L+1(+26%)
	329.4/354.2	3.76/3.50	0.1089/0.0513	H-0→L+4(+28%)/H-0→L+5(+18%)
5TPA/5TPAM	384.8/403.8	3.22/3.07	0.3650/0.2939	H-0→L+0(+95%)/H-0→L+0(+97%)
	376.6/394.3	3.29/3.14	0.2934/0.1857	H-1→L+0(+81%)/H-1→L+0(+91%)
	327.7/333.1	3.78/3.72	0.2205/0.1851	H-1→L+2(+73%)/H-1→L+5(+30%)

The addition of methoxy groups in the structure (TPAM) produced a bathochromic shift in the absorption spectra of all compounds. See Figures 5A,B and Table 3. The main electronic transition for almost all the derivatives was H-0→L+0 corresponding to the maximum absorption wavelength, except for the anthracene derivative, which showed an electronic transition of H-0→L+3 (3TPA in 327.6 nm) and H-0→L+4 (3TPAM in 339.2 nm) for λ_max_, nevertheless, the H-0→L+0 electronic transition was observed in the bands 427.2 nm (3TPA) and 450.5 nm (3TPAM) located in the visible region of the spectra. The H-0→L+0 transition corresponds to the lowest vertical absorption energy (Ω_A_). On the other hand, the pyrrole derivatives show the same behavior as the anthracene derivatives, since they presented electronic transitions of H-0→L+1 (4TPA in 373.3 nm) and H-0→L+2 (4TPAM in 380.0 nm), while the H-0→L+0 transition was not observed in any other band.

### Molecular orbitals and energy levels

**FIGURE 6:**
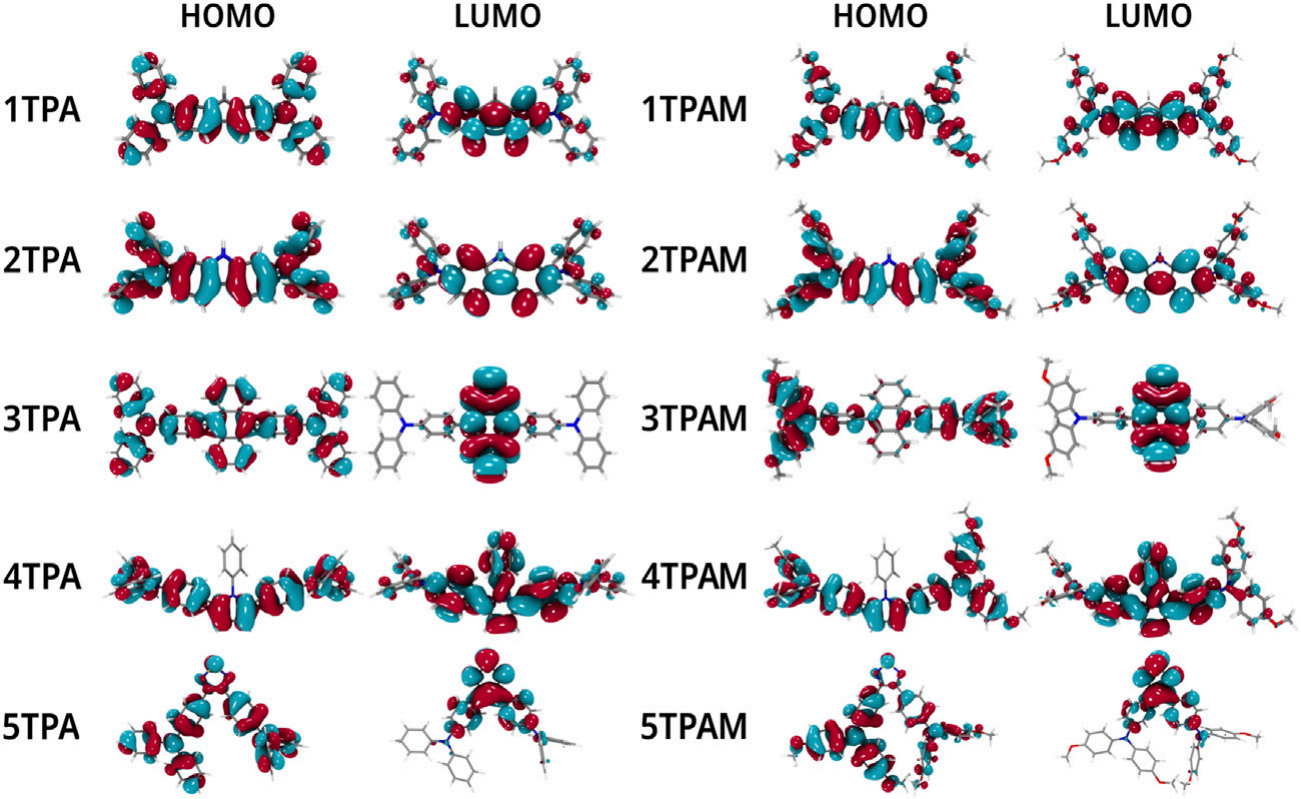
Localization of the frontier molecular orbitals calculated with M06/6-311++G(d,p) in TPA and TPAM derivatives.

The relationship between the optical properties and the electronic structure of the compounds was analyzed by the position of the HOMOs and LUMOs orbitals (See Figure 6). In all the compounds the HOMO orbital is located over the entire molecule, which favors the holes transport (Zhang et al., 2017; Hussain et al., 2021), since the more delocalized molecular orbitals the faster charge transport, due to the reduction of the nuclear reorganization energy (Xu et al., 2018). On the other hand, the LUMO orbital is mainly concentrated in the π-linker zone, except for anthracene and pyrrole derivatives. When a methoxy group was added, only 3TPAM and 5TPAM derivatives showed a change in the HOMO distribution (See Figure 6). The methoxy groups substituted in the phenolic rings act as electron donor groups, causing the HOMO orbital extends slightly towards the periphery of the molecule.

**FIGURE 7:**
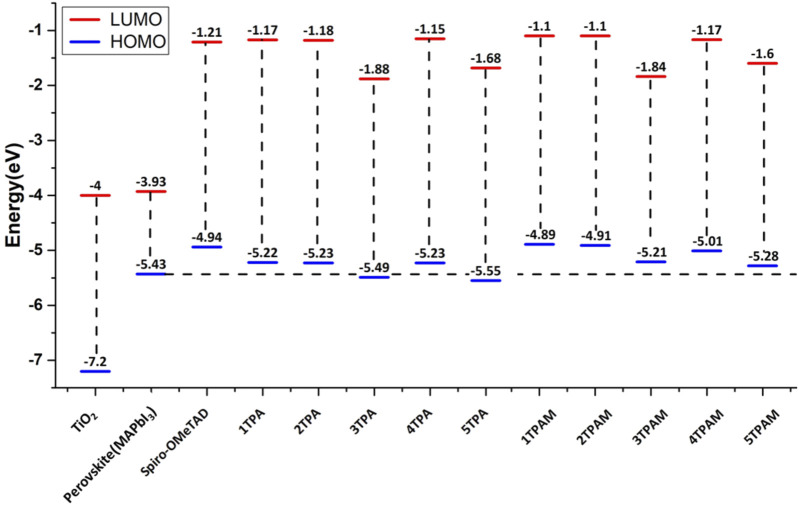
Comparison of energy levels of TPA and TPAM derivatives calculated with M06/6-311++G(d,p).

The energy levels of HOMOs were compared and analyzed with the valence band of the perovskite since a good alignment improves the charge transport processes that occur in a PSC. The HOMO of the HTM should be located above the valence band of the perovskite, which provides the driven force to promote holes transport, and the LUMO should be positioned at a level higher than the conduction band of the light-sensitive material, thus preventing charge recombination. Figure 7 shows the calculated energy levels of the TPA derivatives and these are compared with the energy levels of perovskite and Spiro-OMeTAD, which is taken as HTM reference. The HOMO of almost all the derivatives is above the valence band of the perovskite, except 3TPA and 5TPA, therefore, these two derivatives could not provide the driven force to promote holes transport. The other derivatives could provide the necessary driving force to conduct electrons and holes to the perovskite at the same time. In addition, all the compounds presented HOMO energy values lower than −4.94 eV (Spiro-OMeTAD HOMO energy value); therefore, the new 4TPA could provide a better capacity to conduct holes in comparison with Spiro-OMeTAD, due to the shorter difference between the valence band of the traditional perovskite and the HOMO energy value of our proposed new HTM. Furthermore, the electron hopping from the perovskite to the metal electrode could be efficiently blocked in all derivatives, since the LUMO levels are much higher than the conduction band of the perovskite (Zhang et al., 2021b; Cheng et al., 2022). See Figure 7.

The calculated energy levels for the triphenylamine derivatives with R = OMe are shown in Figure 7 Similarly, these values were compared with the energy levels of the perovskite and the Spiro-OMeTAD. The molecules with methoxy groups also presented HOMO energy levels higher than the conduction band of the perovskite. In addition, the substitution of methoxy groups in 3TPA and 5TPA improves the HOMO energy, placing it above the perovskite valence band. Consequently, the HOMO of almost all the compounds was positioned above the HOMO of the Spiro-OMeTAD, with the exception of 3TPAM, 4TPAM, and 5TPAM, potentially converting them into hole transport materials. As can be observed, the substitution with methoxy groups produced a favorable displacement of the energy levels in comparison with the TPA derivatives, which could decrease the possible charge recombination process.

### Chemical reactivity

The stability of the organic compounds is a vital factor that must be taken into account. One of the main problems of the perovskite solar cell is that it degrades rapidly even in low humidity conditions (Lu et al., 2021; Mazumdar et al., 2021). This is due to the high solubility of the perovskite. Some of the solutions proposed to solve this problem are to replace the methylammonium group of the perovskite with a more stable group in humid conditions or to process the perovskite in an inert gas environment. Also, the holes conducting solid materials have an important role in controlling the stability of the cell. Chemical reactivity parameters such as hardness (η), ionization potential (IP), and electron affinity (EA) can be used for the evaluation of the chemical stability in organic compounds. In the same way, photo-induced oxidation can be evaluated by the energy difference between the cation and the neutral molecule. For example, the higher ionization potential value, the higher stability of photo-oxidation.

Table 4 shows the calculated chemical reactivity parameters for the triphenylamine derivatives, these values were compared with the corresponding Spiro-OMeTAD parameters, which were calculated at the same level of theory. As can be observed, almost all the compounds presented IP values greater than the corresponding value of Spiro-OMeTAD (4.85 eV), except for the 1TPAM and 2TPAM, which presented IP values of 4.79 and 4.82 eV, respectively. Therefore, due to the obtained results, it is expected that these new HTMs will be more stable against oxidation caused by the incidence of light, in particular, the 5TPA thiadiazole derivative, since this compound possesses the highest IP value of the analyzed compounds. Similarly, it was also observed that the addition of the methoxy groups produced a decrease in the ionization potential.

**TABLE 4:** Chemical reactivity parameters of the triphenylamine derivatives and Spiro-OMeTAD calculated with M06/6-311++G(d,p).

Compound	IP (eV)	EA (eV)	η (eV)
1TPA	5.11	1.30	1.9
2TPA	5.12	1.32	1.90
3TPA	5.39	2.06	1.67
4TPA	5.12	1.16	1.98
5TPA	5.45	1.73	1.86
1TPAM	4.79	1.14	1.83
2TPAM	4.82	1.17	1.82
3TPAM	5.13	2.02	1.56
4TPAM	4.92	1.04	1.94
5TPAM	5.18	1.65	1.77
Spiro-OMeTAD	4.85	1.33	1.76

The stability of a chemical species is evaluated by absolute hardness (η). This parameter is defined as the resistance of the chemical potential to a change in the electronic configuration. The greater the value of the hardness, the more stable a chemical compound will be (Coppola et al., 2022). Nearly all compounds showed hardness values higher than the corresponding value of Spiro-OMeTAD (1.76 eV), excluding the anthracene derivatives 3TPA and 3TPAM, which presented the lower values. On other hand, the 4TPA derivative presented the highest value of chemical hardness (1.98 eV), and then this compound is expected to present greater stability.

Concerning the electron affinity (EA), high values are characteristic for n-type semiconductors materials (Chai et al., 2011, in the opposite case, low values will be characteristic of hole-conducting materials. As can be seen in Table 4, only the thiadiazole and anthracene derivatives with and without methoxy groups in the structure showed higher values than Spiro-OMeTAD electron affinity value (1.33 eV). However, this parameter does not appear to be very relevant to determine if a compound could be better holes or electrons conductor.

### Reorganization energy

The hole transport reorganization energy $\left(\lambda_{h}\right)$ is another important parameter to be characterized for HTMs. In this section, a comparison between this value and the electron transport reorganization energy $\left(\lambda_{e}\right)$ is presented to determine which charge transfer process requires less energy and, therefore, would dominate over the other.

The reorganization energy for hole and electron transport of the triphenylamine derivatives (TPA and TPAM) is presented in Table 5. In almost all compounds, the hole transport reorganization energy showed lower values than the electron transport reorganization energy ( $\lambda_{h}<\lambda_{e}$ ), consequently, these derivatives could be mainly hole conductors. On the opposite, the 3TPA anthracene derivative seems to be principally an electron conductor, since $\lambda_{h}<\lambda_{e}$ .

**TABLE 5:** Reorganization energies and electronic coupling values calculated for triphenylamine derivatives and Spiro-OMeTAD.

Compound	$\lambda_{h}\left(meV\right)$	$\lambda_{e}\left(meV\right)$	$J_{AB}^{eff}$ (h) (meV)	$J_{AB}^{eff}$ (e) (meV)
1TPA	367.64	418.98	88.64	8.76
2TPA	351.30	390.66	6.47	41.61
3TPA	441.16	417.28	36.04	96.44
4TPA	544.12	574.69	34.72	24.95
5TPA	103.20	467.42	11.96	89.60
1TPAM	251.75	530.94	79.95	18.69
2TPAM	270.66	463.68	5.99	15.00
3TPAM	197.51	460.84	6.67	95.29
4TPAM	405.57	688.30	10.49	25.17
5TPAM	187.27	564.69	16.80	177.87
Spiro-OMeTAD	163.31	376.11	—	—

These data were compared with the reorganization energy of the Spiro-OMeTAD, calculated at the same level of theory. The thiadiazole derivatives presented a hole transport reorganization energy values similar to those calculated for the Spiro-OMeTAD. As shown in Table 5, the 5TPA, 3TPAM, and 5TPAM derivatives exhibited similar values of hole transport reorganization energy in comparison with the Spiro-OMeTAD, even more, the 5TPA thiadiazole derivative presented lower reorganization energy (103.20 meV) than the Spiro-OMeTAD (163.31 meV). This indicates that the 5TPA thiadiazole derivative could be a better hole conductor. On the other hand, although the pyrrole derivatives (4TPA and 4TPAM) presented the highest values for the hole transport reorganization energy, these values are still smaller than their respective electron transport reorganization energies, and thus, these derivatives are expected to behave as hole conductors.

### Intermolecular electronic coupling

Another crucial parameter for the charge transport properties in organic semiconductors is the electronic coupling. The combination of low reorganization energies and high electronic couplings can maximize the carrier transport rate.

Table 5 shows the calculated electronic couplings for the dimer systems. These values are in agreement with reorganization energy predictions only for 4 molecules: 1TPA, 1TPAM, 4TPA, and 3TPA. While reorganization energy values in monomers exhibited that all the molecules, except 3TPA, are mainly hole conductors, the electronic coupling calculations presented high values for hole transport for 1TPA (88.64 meV), 1TPAM (79.95 meV), and 4TPA (34.72 meV) and, low values for the case of anthracene derivatives (3TPA and 3TPAM). This agrees with the prediction of reorganization energy values which showed higher tendency for the electron transport than for the hole transport in the anthracene derivatives.

**FIGURE 8:**
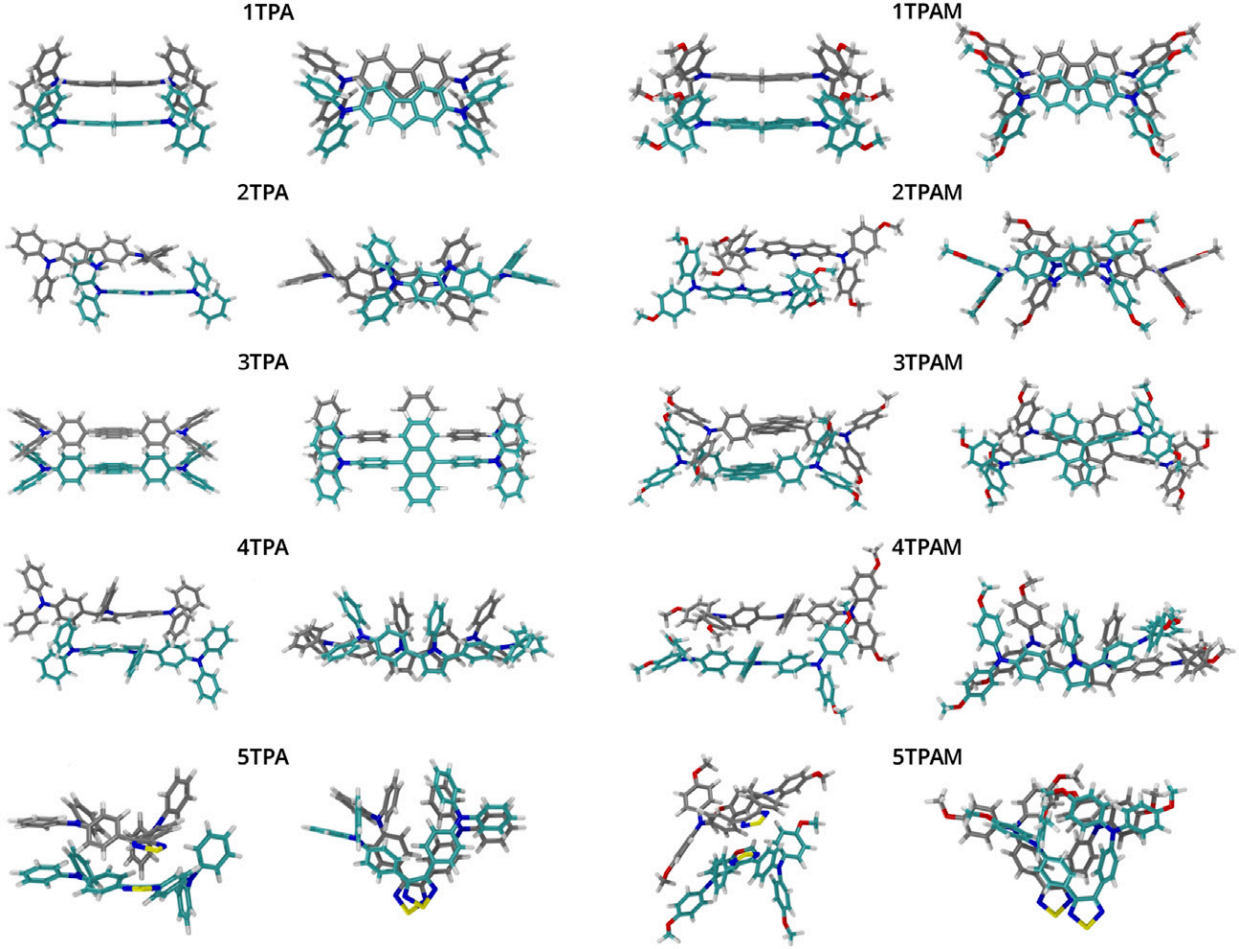
Optimized geometrical structures of the dimers obtained with B3LYP/6-311+G(d).

A detailed structural analysis of the monomers and dimers provided valuable information to understand the consistency in these parameters for the above mentioned molecules. As will be shown, the dimers with less deformation and with a π-π stacking, presented the best agreement between reorganization energy and electronic coupling predictions. Figures 3, 8 allow to identify qualitatively those dimers with less deformation. In addition, values of the root mean squared displacement (RMSD) calculated between each molecule forming the dimer and the optimized monomeric structure (See Supplementary Table S1), showed that the structures of 1TPA, 1TPAM, and 3TPA were those with lowest deformations, followed by 2TPA and 4TPA. However, even though the RMSD values of 2TPA are lower than those for 4TPA, in 2TPA the interaction of the triphenylamine rings causes a rotation of one monomer around its longitudinal axis, causing the π-π interaction to decrease. The effect of a geometric change can also be noted on the site energies of monomers A and B shown in Supplementary Table S2, where the largest difference in this energy occurs in those molecules with the largest RMSD. It has recently been reported (Blaskovits et al., 2021), that flexible molecules can present a wide range of reorganization energies, strongly dependent on their geometry. And, since this geometry is affected by the restrictions imposed due to the interactions with neighbor molecules, the approach of computing *λ* from a lowest energy conformer in the gas phase is not appropriate for flexible structures. This could be the reason for the disagreement between the reorganization energy and electronic coupling for some of the molecules.

**FIGURE 9:**
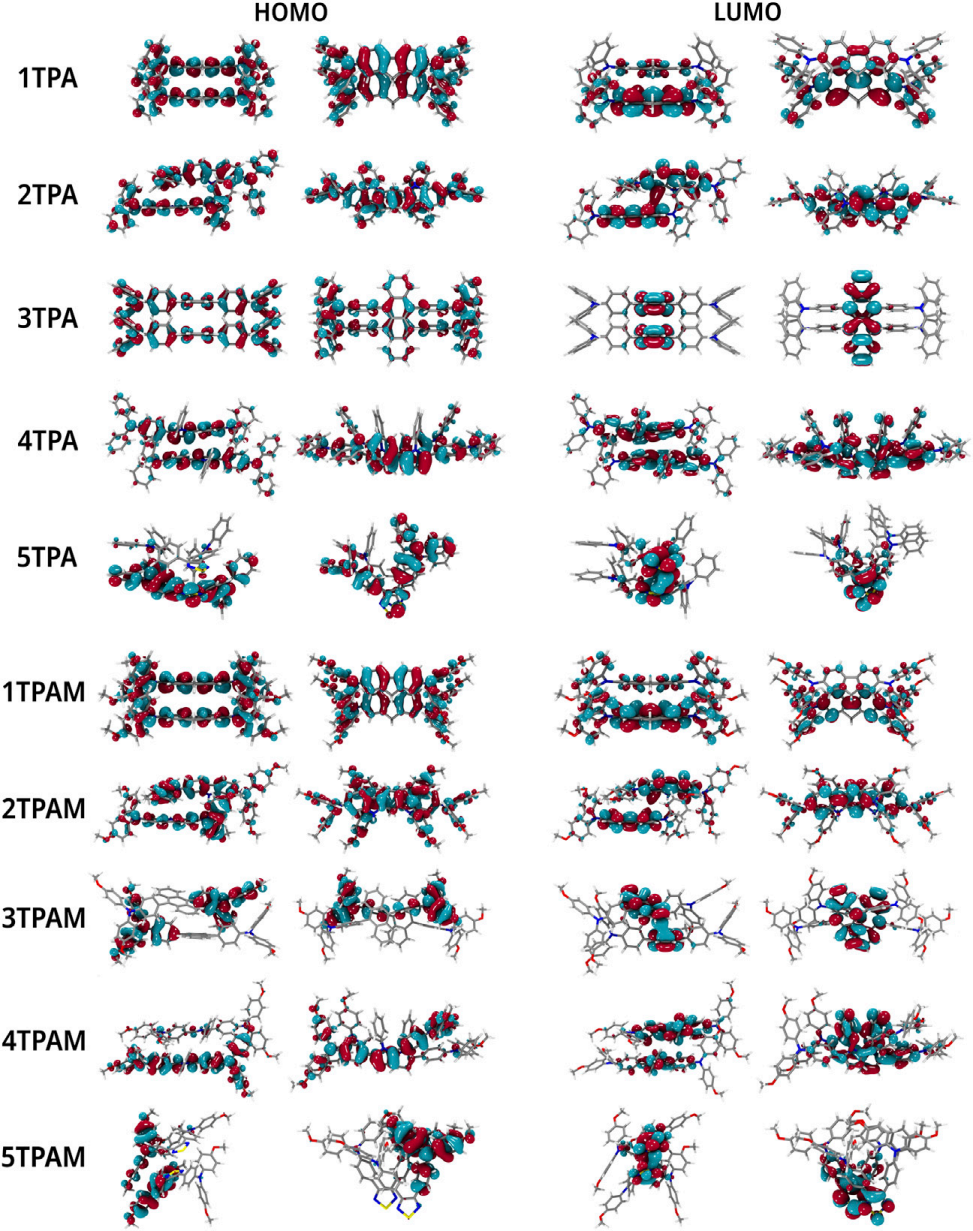
Localization of the frontier molecular orbitals in dimers of TPA and TPAM.

The HOMO and LUMO topologies of monomers and dimers are shown in Figures 6, 9, respectively. As can be observed, when the dimer is formed by 1TPA and 1TPAM, the shape of the HOMO wave function in the dimer is almost the same compared to that in the monomer. On the opposite, the LUMO wave function is mainly concentrated in one molecule of the dimer, making the LUMO overlap ( $S_{AB^{L\;}}$ ) an order of magnitude lower than the HOMO overlap (See Supplementary Table S2). Therefore, 1TPA and 1TPAM will be better hole conductors than electron conductors. The same explanation can be done for 4TPA, however, in this case, the difference in the overlap values was much smaller, causing the electronic coupling values ( $J_{AB}^{eff\;}$ ) for hole and electron conduction be closer than those calculated for 1TPA and 1TPAM.

The dimers with the highest electronic coupling values for hole transport were 1TPA and 1TPAM, with 88.64 and 79.95 meV, respectively, while for electron transport, the highest values were obtained for 5TPAM, 3TPA, 3TPAM, and 5TPA with 177.87, 96.49, 95.29, and 89.60 meV, respectively. In fact, for the molecules studied in this work, if the dimer is a better hole or electron conductor, can be inferred from the spatial overlap values, $S_{AB}$ , shown in Supplementary Table S2. Dimers with HOMO overlap higher than LUMO overlap are better hole conductors and vice versa.

In the new proposed HTMs, 4TPA and 4TPAM (pyrrole derivatives), the electronic coupling values for hole transport were 34.72 and 10.49 meV, respectively; while for 5TPA and 5TPAM (thiadiazole derivatives) the calculated values were 11.96 and 16.80 meV, respectively. Based on these results, 4TPA could be a hole conductor material, whereas the 4TPAM, 5TPA, and 5TPAM seem to be electron conductor materials. After a comparison with fluorene and carbazole derivatives, it can be concluded that 4TPA presented a better hole conducting capacity than carbazole derivatives. Amongst all the dimers, 5TPA and 5TPAM showed the lowest reorganization energies, which combined with a reasonable electronic coupling value, could result in a good carrier transport rate.

In summary, the quantum-chemical calculations presented in this study are useful to understand the relationship between the chemical structure and the electronic properties in the studied molecules. In this case, the molecular structure analysis using different π-linkers revealed that these influence the planarity of the structures which could give more uniformity to the electronic density, thus favoring the charge transport process in the molecules. Additionally, the analysis of chemical reactivity parameters such as hardness (η), ionization potential (IP), and electron affinity (EA) were used for the study of the chemical stability in the analyzed organic compounds. Also, the examination of the molecular orbitals positions, energy levels, electronic transitions, and reorganization energies were extremely helpful to understand the relationship between the electronic structure and the optical properties of the studied molecules. The dimer construction for each molecule was necessary to make a detailed analysis of the charge transfer capacity; being the reorganization energies and the intermolecular electronic coupling the key parameters to define the charge transport capacity of a material.

## Conclusion

New HTMs with triphenylamine units were theoretically analyzed using DFT, TD-DFT and DFTB calculations. The influence of different π-linkers on the absorption spectrum, frontier orbitals, chemical reactivity parameters, reorganization energy and intermolecular electronic coupling were studied. The obtained results suggest that all the compounds absorb energy in the ultraviolet range, which is favorable for an HTM. In addition, almost all the triphenylamine derivatives presented a HOMO energy level that is favorably aligned with the perovskite valence band, except 3TPA and 5TPA. In comparison with the Spiro-OMeTAD, practically all the derivatives showed lower values than the Spiro-OMeTAD HOMO, except 1TPAM and 2TPAM.

Regarding the new proposals, the reorganization energy values suggested that both, pyrrole and thiadiazole derivatives, could be hole transport materials. The 5TPA (thiadiazole derivative) exhibited the lowest hole transport reorganization energy (103.20 meV), being even lower than the reported value for the Spiro-OMeTAD (163.3 meV).

The analysis of the intermolecular electronic coupling carried out on dimers showed different trends compared to the reorganization energy values, which could be due to the flexibility of the molecules. However, in 4TPA derivative both values were consistent with the hole conducting capacity of this material. However, these properties would be better described considering the organic molecules in a single crystal, where there would be more conduction channels.

## Data Availability

The original contributions presented in the study are included in the article/Supplementary Material, further inquiries can be directed to the corresponding author.
