# Supplementary material for: Design of new hole transport materials based on triphenylamine derivatives using different π-linkers for the application in perovskite solar cells. A theoretical study
Source: Front Chem. 2022 Aug 5;10:907556. doi: 10.3389/fchem.2022.907556 (PMC9389019; doi:10.3389/fchem.2022.907556)
Supplement: Supplementary file 1 [file DataSheet1.docx]

Supplementary Material


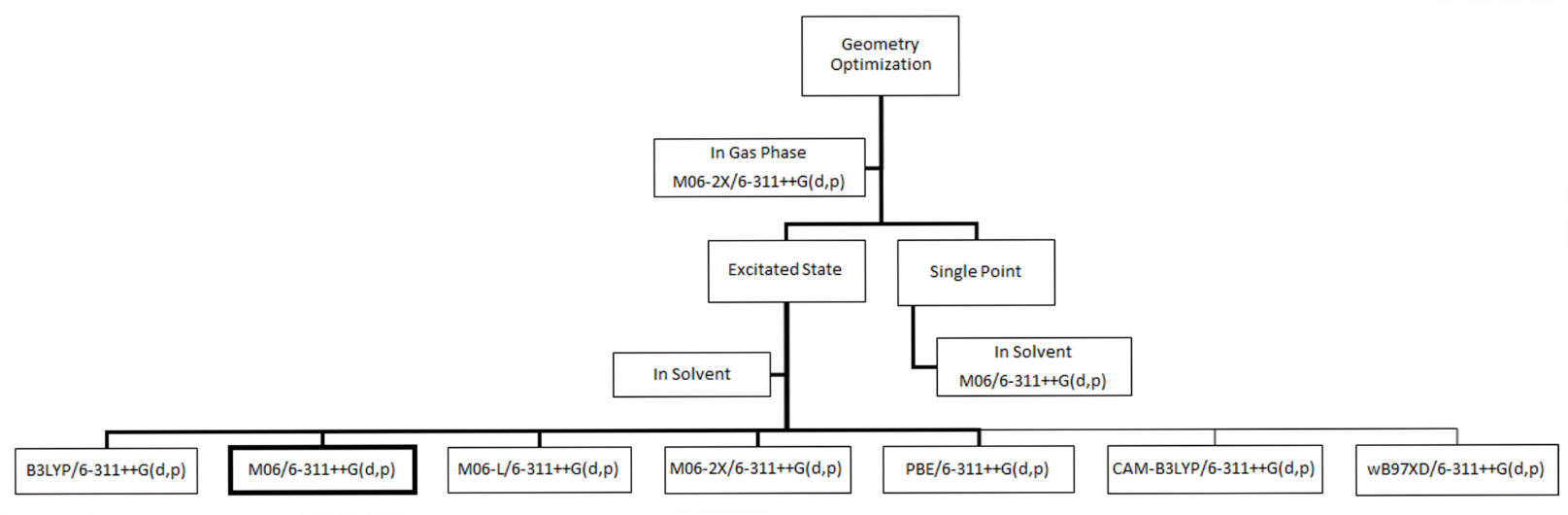


**Scheme 1.** Theoretical methodology used in geometry optimization and electronic properties calculations.

| **Table S1.** Root-Mean Squared Deviation (RMSD) for every conformer analyzed. Mol A and Mol B, are the molecules composing each conformer. | | |
| --- | --- | --- |
| **Compound** | | **RMSD (Å)** |
| 1TPA | Mol A | 0.216 |
|  | Mol B | 0.110 |
| 2TPA | Mol A | 0.264 |
|  | Mol B | 0.384 |
| 3TPA | Mol A | 0.131 |
|  | Mol B | 0.377 |
| 4TPA | Mol A | 0.298 |
|  | Mol B | 0.648 |
| 5TPA | Mol A | 0.991 |
|  | Mol B | 1.108 |
| 1TPAM | Mol A | 0.242 |
|  | Mol B | 0.216 |
| 2TPAM | Mol A | 0.447 |
|  | Mol B | 0.732 |
| 3TPAM | Mol A | 0.974 |
|  | Mol B | 0.710 |
| 4TPAM | Mol A | 0.219 |
|  | Mol B | 0.783 |
| 5TPAM | Mol A | 1.028 |
|  | Mol B | 1.147 |

| **Table S2.** Electron coupling $J_{AB}^{eff}$ ((h) for holes and (e) for electrons), $e_{AB}^{eff}$ site energies (monomer A and monomer B), S_AB_ spatial overlap integral, for triphenylamine derivatives. | | | | | | | | |
| --- | --- | --- | --- | --- | --- | --- | --- | --- |
| **Compound** | $J_{AB}^{eff}$**(h) (meV)** | $J_{AB}^{eff}$**(e) (meV)** | $\boldsymbol{e}_{\boldsymbol{A}}^{\boldsymbol{eff}}$**(h) (eV)** | $\boldsymbol{e}_{\boldsymbol{B}}^{\boldsymbol{eff}}$ **(h) (eV)** | $\boldsymbol{e}_{\boldsymbol{A}}^{\boldsymbol{eff}}$**(e) (eV)** | $\boldsymbol{e}_{\boldsymbol{B}}^{\boldsymbol{eff}}$ **(e) (eV)** | **S_AB_(h)** | **S_AB_(e)** |
| 1TPA | 88.642 | 8.766 | -4.801 | -4.819 | -1.235 | -1.308 | -0.0164 | -0.0037 |
| 2TPA | 6.477 | 41.615 | -4.940 | -4.940 | -1.485 | -1.485 | -0.0007 | 0.0141 |
| 3TPA | 36.049 | 96.448 | -5.106 | -5.106 | -1.986 | -1.986 | -0.0360 | -0.0219 |
| 4TPA | 34.723 | 24.951 | -4.665 | -4.665 | -1.233 | -1.233 | 0.0050 | 0.0042 |
| 5TPA | 11.963 | 89.603 | -5.312 | -5.291 | -2.046 | -2.026 | 0.0033 | 0.0181 |
| 1TPAM | 79.95 | 18.69 | -4.407 | -4.436 | -0.982 | -1.082 | 0.0143 | 0.0040 |
| 2TPAM | 5.994 | 15.003 | -4.496 | -4.496 | -1.214 | -1.214 | -0.0000 | 0.0098 |
| 3TPAM | 6.679 | 95.298 | -4.822 | -4.822 | -1.814 | -1.814 | -0.0014 | -0.0212 |
| 4TPAM | 10.495 | 25.178 | -4.544 | -4.517 | -1.096 | -1.089 | 0.0015 | -0.0106 |
| 5TPAM | 16.809 | 177.870 | -4.945 | -4.935 | -2.112 | -1.876 | 0.0020 | 0.0307 |
